# Histologic Assessment of a Fast-Set Mineral Trioxide Aggregate (MTA) and Two Novel Antibacterial-Enhanced Fast-Set MTAs for Apexification and Periapical Healing of Teeth With Incomplete Root Formation in a Rat Model: An In Vivo Animal Study

**DOI:** 10.7759/cureus.59064

**Published:** 2024-04-26

**Authors:** Ayesha Fathima, Vignesh Ravindran, Ganesh Jeevanandan, Karthik Ganesh Mohanraj, Rajalakshmanan Eswaramoorthy, Abirami Arthanari

**Affiliations:** 1 Department of Pediatric and Preventive Dentistry, Saveetha Dental College and Hospitals, Saveetha Institute of Medical and Technical Sciences, Saveetha University, Chennai, IND; 2 Department of Anatomy, Saveetha Dental College and Hospitals, Saveetha Institute of Medical and Technical Sciences, Saveetha University, Chennai, IND; 3 Center of Molecular Medicine and Diagnostics, Department of Biochemistry, Saveetha Dental College and Hospitals, Saveetha Institute of Medical and Technical Sciences, Saveetha University, Chennai, IND; 4 Department of Forensic Odontology, Saveetha Dental College and Hospitals, Saveetha Institute of Medical and Technical Sciences, Saveetha University, Chennai, IND

**Keywords:** cytotoxicity, calcium fluoride, doxycycline, metronidazole, calcific barrier

## Abstract

Background

Pulp necrosis in incomplete root formation halts dentine development, resulting in larger canals with fragile walls and an open apex, complicating canal instrumentation and apical stop formation. Bioactive endodontic cements such as mineral trioxide aggregate (MTA) are crucial for creating artificial apical barriers or inducing apical foramen closure, but challenges remain regarding their antimicrobial efficacy and cytotoxicity. Modifications to MTA formulations aim to address these concerns.

Methods

This in vivo animal study involved 80 Wistar albino rats, with incomplete root formation induced by pulp exposure. Rats were divided into four groups receiving different MTA formulations for apexification: conventional MTA, modified MTA, and MTA enhanced with metronidazole or doxycycline. Histopathological evaluations were conducted at seven and 28 days post-treatment to assess calcific barrier formation, inflammatory reactions, and antimicrobial efficacy.

Results

By day 7, modified MTA formulations exhibited enhanced antibacterial activity compared to conventional MTA (p = 0.000), with fewer inflammatory reactions and microorganisms. By day 28, modified formulations showed superior calcific barrier formation, particularly in the metronidazole- and doxycycline-enhanced groups compared to conventional MTA (p = 0.000). These outcomes suggest that modifications to MTA formulations improve antimicrobial efficacy and calcific barrier formation in vivo.

Conclusion

Novel modified MTA formulations, particularly those enhanced with metronidazole or doxycycline, exhibit superior antibacterial efficacy and calcific barrier formation compared to conventional MTA. Further long-term studies are warranted to validate these findings for potential clinical translation.

## Introduction

Pulp necrosis before complete root formation halts dentine formation and also ceases root development. This leads to a larger canal with fragile walls and an open apex, making canal instrumentation challenging and hindering adequate apical stop formation. To facilitate root-filling material condensation and ensure an effective apical seal, creating an artificial apical barrier or inducing apical foramen closure is necessary [1,2].

Bioactive endodontic cements are materials with the capability to form apatite in body fluids and are utilized for a variety of dental procedures such as pulp capping and root canal treatments [3]. Despite differences in composition, these materials exhibit similar bioactive properties. The commonly employed bioactive endodontic cements include calcium-based materials, mineral trioxide aggregate (MTA), and biodentine. Among these, MTAs are widely favored due to their high biocompatibility and effectiveness in sealing and treating root canals [4]. MTAs comprise calcium silicates and minor amounts of other calcium compounds. Different brands of MTA, such as ProRoot MTA, Angelus MTA, and MTA Plus, offer various options for clinical use. However, the diversity of available bioactive materials necessitates clear guidance for their appropriate application in different clinical scenarios. Despite MTAs' advantages, issues such as cost, setting time, and tooth discoloration have prompted the introduction of newer bioactive endodontic cements to the market [3].

*Enterococcus faecalis* stands out as the most commonly encountered bacterium in stubborn infections post-endodontic therapy [5]. Prior investigations have yielded contradictory findings concerning MTA's ability to combat *E. faecalis*. Some studies indicate that MTA falls short in restraining the proliferation of *E. faecalis* [6]. However, contrasting research suggests that MTA does indeed exhibit antibacterial properties against *E. faecalis* [7]. Efforts to boost the antimicrobial efficacy of mineral trioxide aggregate (MTA) by integrating agents such as chlorhexidine gluconate and tetracycline are significant. However, it is crucial to strike a balance between enhancing antimicrobial properties and preserving the cement's desired physical characteristics. Challenges arise from potential compromises to the cement's physical properties when incorporating antimicrobial agents, necessitating thorough evaluations [8]. Furthermore, attempts to reduce MTA's cytotoxicity and enhance handling, sealing ability, and biocompatibility are essential. MTA's ability to stimulate dentin formation is valuable for various pulp therapies. Recognizing the cytotoxic properties of tricalcium aluminate in traditional MTA is critical for long-term treatment assessment [9]. Addressing these issues is vital for maintaining the cement's biocompatibility, which is crucial for dental practitioners.

The authors have attempted to improvise these drawbacks with the addition of calcium fluoride (enhanced antibacterial action) [5] and calcium carbonate (enhanced calcification) [10] and the elimination of tricalcium aluminate (cytotoxic effect) [9] and have concluded with better results in regard to antimicrobial properties [11], compressive strength [12], pushout bond strength [13], and cytotoxic properties [14]. Such new materials have to be tested in animal models before their clinical application in humans. Hence, this study aimed to evaluate the effect of modified and antibacterial-enhanced MTAs on apexification and periapical healing in a rat model with incomplete root formation.

## Materials and methods

Study design and ethical clearance

The design of this in vivo animal research was approved by the Scientific Review Board of the institute. The research methodology was discussed with the Institutional Animal Ethics Committee and approved (reference number: BRULAC/SDCH/SIMATS/IAEC/09-2023/01).

Sample size

The sample size was determined based on a previously published animal research study [1], aiming for a study power of 80% and a confidence level of 95%. Utilizing an α value of 0.05, an effect size of 0.45, and a desired power of 0.95, it was determined that a minimum sample size of 35 teeth per group owing to 140 teeth would be necessary to conclude its results with superior quality.

Study animals

Eighty rats of the Wistar albino lineage (*Rattus norvegicus*), weighing between 150 and 200 g and approximately four weeks old, were obtained from the Biomedical Research Unit and Lab Animal Centre housed within the institute. The rats were enclosed in a chamber with a stable temperature of 25°C ± 1°C and subjected to a 12-hour light and dark cycle. They had unrestricted access to rat pellet feed (Biogen Animal Health, Bangalore, India) and water.

Preoperative preparation

The handling of the rats, treatment procedure, maintenance during the observation period, and euthanasia of the animals were done by a single trained veterinary specialist with the guidance of the authors for dental-related procedures. All the rats were anesthetized intraperitoneally with ketamine 50 mg/kg (Imalgene® 1000, Merial, Lyon, France) and xylazine 10 mg/kg (Rompun® 2%, Bayer, Leverkusen, Germany). A retractor-like device was used to keep the rats' mouths open during the experimental procedures. Access opening was done using ¼ carbide round bur on the cingulum region of both the maxillary central incisors. The pulp tissues were completely removed with a #8 followed by #10 K-file (Mani, Tochigi, Japan), followed by irrigation using saline. The canals were left open without coronal filling for three weeks to induce pulp necrosis.

Treatment protocol

The canals were irrigated with saline to remove any food debris, followed by confirmation of working length using an electronic apex locator (Root ZX mini, J Morita, Tokyo, Japan). The canal was prepared using #15, #20, and #25 K-file (Mani). With every increase in file, the canal was irrigated with saline and 1% sodium hypochlorite. Final irrigation was performed using saline before placement of the apexification material. Forty rats were treated using conventional MTA, MTA Angelus (Londrina, Brazil) (Group I) in the right maxillary central incisors (40 teeth) and a novel modified MTA formulation (Group II) in the left maxillary central incisors (40 teeth). The remaining 40 rats were treated using modified MTA enhanced with metronidazole (Group III) in the right maxillary central incisors (40 teeth) and modified MTA enhanced with doxycycline (Group IV) in the left maxillary central incisors (40 teeth). Therefore, a total of 160 teeth were treated with the test materials. The composition of the test materials is described in Table 1.

**Table 1:** Composition of powder contents of the modified mineral trioxide aggregate and the two antibacterial-enhanced mineral trioxide aggregates used in the current study MTA: mineral trioxide aggregate

Group II (modified MTA formulation)		Group III (metronidazole-enhanced MTA)		Group IV (doxycycline-enhanced MTA)	
Powder	Weight % for every 100 mg of powder	Powder	Weight % for every 100 mg of powder	Powder	Weight % for every 100 mg of powder
Tricalcium silicate	55 wt %	Tricalcium silicate	60 wt %	Tricalcium silicate	60 wt %
Dicalcium silicate	30 wt %	Dicalcium silicate	20 wt %	Dicalcium silicate	20 wt %
Calcium fluoride	5 wt %	Calcium fluoride	5 wt %	Calcium fluoride	5 wt %
Calcium sulfate	5 wt %	Calcium sulfate	5 wt %	Calcium sulfate	5 wt %
Calcium carbonate	5 wt %	Calcium carbonate	4 wt %	Calcium carbonate	4 wt %
Zirconium oxide	1 wt %	Metronidazole	5 wt %	Doxycycline	5 wt %
-	-	Zirconium oxide	1 wt %	Zirconium oxide	1 wt %

For the novel MTA groups (Groups II, III, and IV), a blend of 100 mg of the specified powder content and 40 µL of the liquid (10% calcium chloride) was deposited onto a mixing pad. Following complete hydration, the mixture was thoroughly homogenized until a uniform consistency suitable for molding was attained. For the conventional MTA group, MTA Angelus (Londrina, Brazil) was dispensed as recommended by the manufacturer (3:1 ratio) on a mixing pad. The powder was incrementally added to the liquid until complete hydration and mixed until a thick moldable consistency was obtained. The final mix was carried using a #25 plugger (Mani) and placed at the apex based on the determined working length and reconfirmed using a radiograph. The remaining portion of the canal was filled with mixed material, and coronal filling was done using restorative glass ionomer cement (GC Corporation, Tokyo, Japan). The rats were housed back in their chambers with a routine diet and water ad libitum. Pain management was done by injecting meloxicam (1 mg/kg body weight) twice a day for the first two days only. By the third day, pain management was performed only if the rat was found to be not having its regular meal, suggesting aggravated pain during mastication.

Euthanasia and histopathological processing

Seven rats from each group (28 rats) were sacrificed after seven days, and the remaining 28 rats were sacrificed after 28 days of treatment. The rats were sacrificed by euthanizing them using carbon dioxide gas inhalation in a closed chamber. The jaws containing experimental teeth were collected and fixed in 10% formalin for 24 hours before decalcification with a 17% ethylenediaminetetraacetic acid solution. After processing and embedding in paraffin, they were trimmed until the apical foramen was exposed. Transverse sections with a thickness of 6 µm were obtained and stained with hematoxylin and eosin. Root section images were examined using light microscopy (Eclipse E600; Nikon, Tokyo, Japan) to analyze periodontal ligament, cementum, and dentin characteristics. Digital images were captured using Nikon DXm1200C (Nikon) with fixed magnification (40×) and analyzed for the presence or absence of calcified tissue barrier, inflammatory reaction, resorptive changes of bone or root, and microorganisms.

Statistical analysis

The Statistical Package for the Social Sciences (SPSS) software version 21.0 (IBM SPSS Statistics, Armonk, NY) was used to compare the histologic data that were obtained and tabulated. Analysis of the obtained data was performed using the non-parametric Kruskal-Wallis test, followed by the post hoc Mann-Whitney U test. A p-value of less than 0.05 was considered statistically significant.

## Results

All the study rats tolerated the operative procedures well. Their behavior and eating habits did not change and were not affected by the procedure done. No animal or teeth were lost during the follow-up periods.

Histopathological analysis on the seventh day

By the end of the seventh day, none of the histopathological samples showed signs of calcified tissue barrier, bone resorption, and root resorption. However, there was evident inflammatory reaction in all groups. All samples (100%) from Group I had inflammatory infiltrate, while about 50% of the samples from Group II had inflammatory infiltrate. About 65% of the samples in both Groups III and IV did not have an inflammatory reaction. These results also coincided with the presence or absence of microorganisms, i.e., samples with inflammatory infiltrate had microorganisms. On intergroup comparison, a significant difference was noticed between the groups, showing enhanced antibacterial activity in samples under Groups III and IV (p = 0.000) (Table 2).

**Table 2:** Histopathological analysis of the seventh-day samples across the four groups *Statistically significant for intergroup comparisons (p < 0.05) MTA: mineral trioxide aggregate

Day 7		Group I (conventional MTA)		Group II (novel modified MTA)		Group III (MTA enhanced with metronidazole)		Group IV (MTA enhanced with doxycycline)		p-value
		Number	%	Number	%	Number	%	Number	%	
Calcified tissue barrier	Present	0	0	0	0	0	0	0	0	-
	Absent	20	100	20	100	20	100	20	100	
Inflammatory reaction	Present	20	100	10	50	7	35	7	35	0.000*
	Absent	0	0	10	50	13	65	13	65	
Bone resorption	Present	0	0	0	0	0	0	0	0	-
	Absent	20	100	20	100	20	100	20	100	
Root resorption	Present	0	0	0	0	0	0	0	0	-
	Absent	20	100	20	100	20	100	20	100	
Microorganisms	Present	20	100	10	50	7	35	7	35	0.000*
	Absent	0	0	10	50	13	65	13	65	

**Figure 1:**
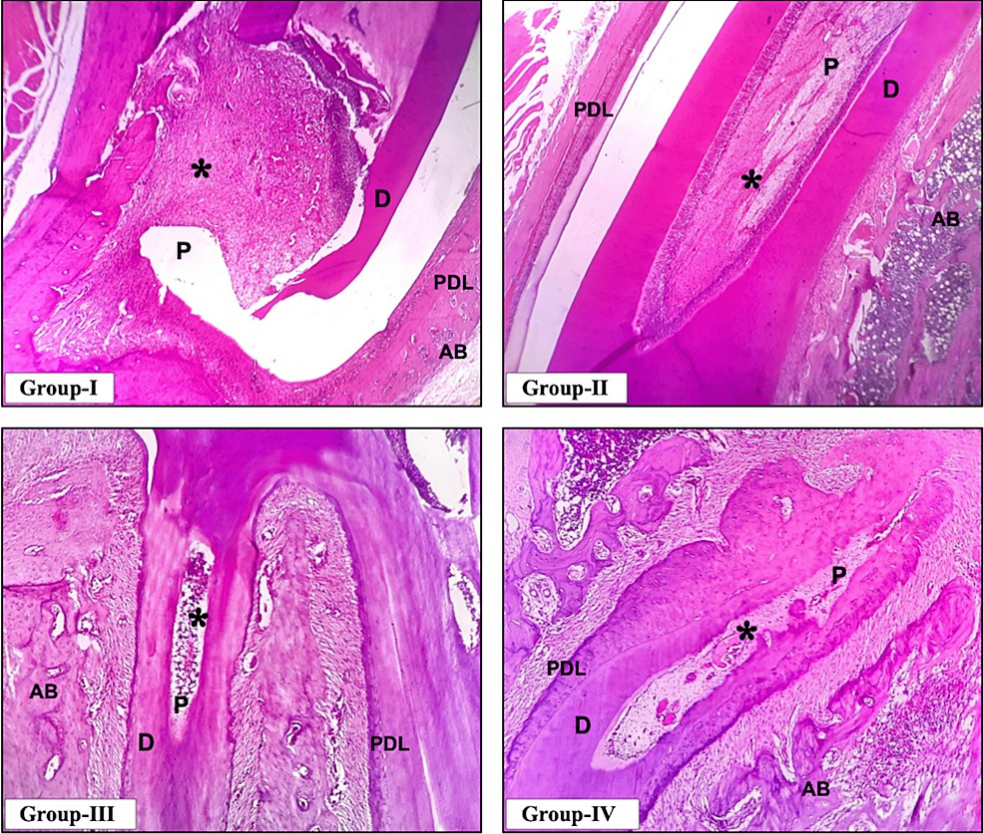
Representative histopathological image of the seventh-day samples across the four groups *: inflammatory cells, AB: alveolar bone, D: dentin, P: pulp, PDL: periodontal ligament

By the end of the seventh day, typical features of inflammation were noticed across all groups, characterized by infiltration of inflammatory cells in the pulp cavity. After the onset of inflammation, by day 7, the inflammatory cells continued to increase in Group I when compared to Groups II, III, and IV. Among Groups II, III, and IV, Group III showed better tissue response and prevented inflammatory reactions, which is evident from the pulp cavity containing reduced inflammatory cells. A representative histopathological image of the majority of samples associated with the respective group is shown in Figure 1.

Histopathological analysis on the 28th day

By the end of the 28th day, none of the histopathological samples showed signs of bone and root resorption. However, there was an evident calcific barrier and inflammatory reactions occurring in all groups. The highest percentage of samples showing calcific barrier formation was noticed in Group II (80%), followed by Groups IV (65%), III (60%), and I (25%). As regards inflammatory reaction, there was the presence of inflammatory cells in about 75% of samples in Group I, followed by Groups II (30%), III (15%), and IV (15%). None of the samples in Groups III and IV showed the presence of microorganisms, while about 40% of samples in Group II and 75% of samples in Group I had microorganisms. On intergroup comparison, a significant difference was noticed between the groups in relation to calcific barrier formation and the presence or absence of microorganisms and inflammatory reactions (p = 0.000) (Table 3).

**Table 3:** Histopathological analysis of the 28th-day samples across the four groups *Statistically significant for intergroup comparisons (p < 0.05) MTA: mineral trioxide aggregate

Day 28		Group I (conventional MTA)		Group II (novel modified MTA)		Group III (MTA enhanced with metronidazole)		Group IV (MTA enhanced with doxycycline)		p-value
		Number	%	Number	%	Number	%	Number	%	
Calcified tissue barrier	Present	5	25	16	80	12	60	13	65	0.000*
	Absent	15	75	4	20	8	40	7	35	
Inflammatory reaction	Present	15	75	6	30	3	15	3	15	0.000*
	Absent	5	25	14	70	17	85	17	85	
Bone resorption	Present	0	0	0	0	0	0	0	0	-
	Absent	20	100	20	100	20	100	20	100	
Root resorption	Present	0	0	0	0	0	0	0	0	-
	Absent	20	100	20	100	20	100	20	100	
Microorganisms	Present	15	75	8	40	0	0	0	0	0.000*
	Absent	5	25	12	60	20	100	20	100	

**Figure 2:**
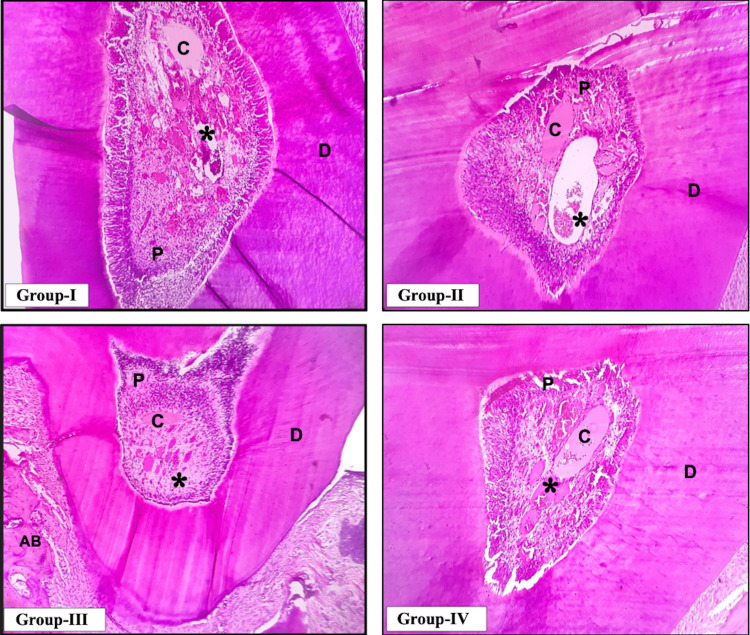
Representative histopathological image of the 28th-day samples across the four groups *: inflammatory cells, AB: alveolar bone, D: dentin, P: pulp, C: calcification for barrier

By the end of the 28th day, the inflammatory cells resolved and reduced across all groups. However, a majority of samples in Group I showed minimal presence of inflammatory cells depicting the continuation of the tissue reaction process when compared to Groups II, III, and IV. As the inflammation subsided gradually by day 28, Group II revealed comparatively better mineralized apical calcific bridge formation than Groups III and IV (Figure 2).

## Discussion

The present study analyzed the histopathological picture of the tissues surrounding the apical region of rat incisors that were treated by apexification using different calcium silicate cements. The newly modified MTA composition had superior calcification potential and moderate antibacterial activity. About half the samples treated with modified MTA had inflammatory infiltration with the presence of microorganisms by the end of the seventh day. However, by the end of the 28th day, about 30% of the samples still had microorganisms and inflammatory reactions. This could be due to the presence of calcium silicates, which enhanced the formation of calcium hydroxide that had an acidic pH causing a bactericidal effect [15]. Also, the addition of calcium fluoride enhances the antimicrobial action of the cement [5].

Metronidazole- and doxycycline-enhanced MTAs performed better with escalated antibacterial activity and justifiable calcification ability. The majority of the samples had an absence of inflammatory reactions and microorganisms by the end of the seventh day and 28th day. This could be due to the presence of calcium silicates and calcium fluoride as suggested earlier. The presence of antibacterial agents enhanced the antimicrobial activity of the final cement [16]. Antibiotics play a crucial role in dentistry, serving as disinfectants in various forms such as irrigants, intracanal medicaments, and additives to dental cements. Nitroimidazole antibiotics such as metronidazole and the tetracycline group, including minocycline and doxycycline, have shown efficacy against *E. faecalis*, a resilient endodontic pathogen, by targeting anaerobic microorganisms through different mechanisms [17]. Incorporating metronidazole and doxycycline into the present study yielded superior outcomes compared to conventional MTA, highlighting the potential benefits of antibiotic enhancement in dental materials [13].

As regards to the formation of the calcific barrier, the majority of the samples treated with modified MTA formulation had a calcific barrier by the end of the 28th day. Also, about 60%-65% of the samples treated with antibacterial-enhanced MTAs had calcific barrier formation by the end of the 28th day. This could be due to the addition of calcium sulfates and carbonates that enhance calcium ion release in the environment, enhancing the calcification process. Also, the new formulation could have induced the odontoblastic potential of nearby stem cells, which would have been the reason for enhanced calcification [18]. Calcium additives did help in the calcification process, which was enhanced with the antimicrobial supplement that created a sterile environment for osteoblastic activity [19].

MTA is the preferred treatment for teeth with open apex and pulp necrosis, facilitating apexification. It serves as a bioceramic material providing chemical signals for periapical tissue regeneration and dentin formation. MTA's properties include the release of calcium and hydroxyl ions during hydration, acting as cues to raise pH and promote mineralized tissue growth. Recent study results are consistent with previous findings, showing mineralized tissue formation similar to past reports [20]. Efforts have been made to improve different aspects of MTA, including reducing its toxicity and enhancing handling, sealing, and biocompatibility [13]. MTA's ability to stimulate dentin growth makes it highly valuable for various pulp treatments such as pulp capping and apexification. However, it is important to acknowledge the cytotoxicity of tricalcium aluminate, a key component in traditional MTA, highlighting the need for long-term treatment assessment to maintain biocompatibility [9]. Nevertheless, various modifications are crucial to balance antimicrobial enhancement with preserving MTA's physical properties, emphasizing the need for calcium barrier formation and root end development.

This study is the first study to assess the in vivo effect in a rat model of a new formulation of MTA and two other antibacterial-enhanced MTA. This is also the first study to analyze these materials' responses during apexification protocol in an animal model. There is a lack of similar in vivo animal studies performed to test new combinations of such calcium silicate cements. Prabhakar et al. [21] assessed curcumin and MTA as pulpotomy agents, revealing that both materials led to a gradual reduction in inflammatory response over time. Curcumin showed better maintenance of pulp architecture, while MTA exhibited more consistent dentinal bridge formation. Another similar study was performed in dog pups for apexification using conventional MTA in previously contaminated canals. They reported significant differences in barrier formation and MTA extrusion between the groups, thereby supporting that MTA favored apexification and periapical healing [1].

However, certain limitations have to be addressed. A long-term evaluation of three months could have been performed to further assess any development in root formation. Samples could have been subjected to nano-computed tomography analysis, which could have provided a three-dimensional perspective of the calcified structure. The exact mechanism of calcific bridge formation would require further analysis in terms of cell lines and also by studying its effect in contact with odontoblasts, osteoblasts, cementoblasts, and periapical stem cells. Future studies can be performed bearing the above limitations in mind, which could help in providing a broadened perspective about the newly formulated materials.

## Conclusions

Novel modified formulation of MTA performed better than conventional MTA in terms of superior calcific bridge formation and acceptable antibacterial efficacy in the rat model. Antibacterial-enhanced MTA formulations using metronidazole or doxycycline performed eminently as they formulated a sterile environment with additional calcific bridge formation in the rat model. Further long-term in vivo analysis is required to be clinically accepted for human trials.

## References

[1] Felippe WT, Felippe MC, Rocha MJ (2006). The effect of mineral trioxide aggregate on the apexification and periapical healing of teeth with incomplete root formation. Int Endod J.

[2] Kuga MC, Duarte MA, Sant'anna-Júnior A, Keine KC, Faria G, Dantas AA, Guiotti FA (2014). Effects of calcium hydroxide addition on the physical and chemical properties of a calcium silicate-based sealer. J Appl Oral Sci.

[3] Maru V, Dixit U, Patil RS, Parekh R (2021). Cytotoxicity and bioactivity of mineral trioxide aggregate and bioactive endodontic type cements: a systematic review. Int J Clin Pediatr Dent.

[4] Solanki NP, Venkappa KK, Shah NC (2018). Biocompatibility and sealing ability of mineral trioxide aggregate and biodentine as root-end filling material: a systematic review. J Conserv Dent.

[5] Lim M, Yoo S (2022). The antibacterial activity of mineral trioxide aggregate containing calcium fluoride. J Dent Sci.

[6] Kim RJ, Kim MO, Lee KS, Lee DY, Shin JH (2015). An in vitro evaluation of the antibacterial properties of three mineral trioxide aggregate (MTA) against five oral bacteria. Arch Oral Biol.

[7] Tanomaru-Filho M, Tanomaru JM, Barros DB, Watanabe E, Ito IY (2007). In vitro antimicrobial activity of endodontic sealers, MTA-based cements and Portland cement. J Oral Sci.

[8] Shin JH, Ryu JJ, Lee SH (2021). Antimicrobial activity and biocompatibility of the mixture of mineral trioxide aggregate and nitric oxide-releasing compound. J Dent Sci.

[9] Moon HJ, Lee JH, Kim JH (2018). Reformulated mineral trioxide aggregate components and the assessments for use as future dental regenerative cements. J Tissue Eng.

[10] Holland R, Gomes JE Filho, Cintra LT, Queiroz ÍOA, Estrela C (2017). Factors affecting the periapical healing process of endodontically treated teeth. J Appl Oral Sci.

[11] Ravindran V, Jeevanandan G (2023). Comparative evaluation of the physical and antimicrobial properties of mineral trioxide aggregate, biodentine, and a modified fast-setting mineral trioxide aggregate without tricalcium aluminate: an in vitro study. Cureus.

[12] Ravindran V, Jeevanandan G, Veeraraghavan VP (2023). Comparative evaluation of physical and antimicrobial properties of doxycycline incorporated formulation of mineral trioxide aggregate - an in-vitro study. J Int Dent Med Res.

[13] Merlin AR, Ravindran V, Jeevanandan G (2024). Comparative evaluation of push-out bond strength of conventional mineral trioxide aggregate, biodentine, and two novel antibacterial-enhanced mineral trioxide aggregates. J Contemp Dent Pract.

[14] Chakravorty A, Ravindran V, Jeevanandan G, Arthanari A (2023). The cytotoxic assessment of antibacterial-enhanced mineral trioxide aggregate compared to commercially available bioceramic cements by using methyl-thiazoldiphenyl-tetrazolium (MTT) assay on human dental pulp stem cells: an in vitro study. Cureus.

[15] Reyes-Carmona JF, Felippe MS, Felippe WT (2009). Biomineralization ability and interaction of mineral trioxide aggregate and white portland cement with dentin in a phosphate-containing fluid. J Endod.

[16] Ravindran V, Jeevanandan G, Veeraraghavan VP (2024). Comparative evaluation of physical and antimicrobial properties of metronidazole incorporated formulation of mineral trioxide aggregate - an in-vitro study. J Int Dent Med Res.

[17] Holt DM, Watts JD, Beeson TJ, Kirkpatrick TC, Rutledge RE (2007). The anti-microbial effect against enterococcus faecalis and the compressive strength of two types of mineral trioxide aggregate mixed with sterile water or 2% chlorhexidine liquid. J Endod.

[18] Huang GT (2009). Apexification: the beginning of its end. Int Endod J.

[19] Panda P, Mishra L, Govind S, Panda S, Lapinska B (2022). Clinical outcome and comparison of regenerative and apexification intervention in young immature necrotic teeth-a systematic review and meta-analysis. J Clin Med.

[20] Nagy MM, Tawfik HE, Hashem AA, Abu-Seida AM (2014). Regenerative potential of immature permanent teeth with necrotic pulps after different regenerative protocols. J Endod.

[21] Prabhakar AR, Mandroli PS, Bhat K (2019). Pulpotomy with curcumin: histological comparison with mineral trioxide aggregate in rats. Indian J Dent Res.

